# Prospective review of 30-day morbidity and mortality in a paediatric neurosurgical unit

**DOI:** 10.1007/s00381-017-3358-5

**Published:** 2017-02-28

**Authors:** Emer Campbell, Thomas Beez, Lorraine Todd

**Affiliations:** 1 Department of Paediatric Neurosurgery, Royal Hospital for Children, 1345, Govan Road, Glasgow, Scotland G51 4TF UK; 2 Department of Paediatric Neurosurgery, University Hospital, Dusseldorf, Germany

**Keywords:** Adverse event, Complication, Paediatric, Neurosurgery, Surveillance

## Abstract

**Purpose:**

The purpose of this study is to record the 30-day and inpatient morbidity and mortality in paediatric patients in a tertiary neuroscience centre over a 2-year period. The intentions were to establish the frequency of significant adverse events, review the current published rates of morbidity in paediatric neurosurgical patients and propose three clinical indicators for future comparison.

**Methods:**

All deaths and adverse events were prospectively recorded from 1 January 2014 to 31 December 2015. Each adverse event was categorised, allocated a clinical impact severity score and linked to a neurosurgical procedure wherever possible. Where a patient suffered several adverse events in the same admission, each event was recorded separately. If a patient had been discharged home, an adverse event was recorded if it occurred within 30 days of admission.

**Results:**

Five hundred forty-nine procedures were performed in 287 patients (aged <16 years). One hundred thirty significant adverse events were identified. The following are the three clinical indicators: significant adverse event rate: 111 (20.2%) operations were linked to at least one significant adverse event; unscheduled return to theatre rate: 81 (14.8%) operations were associated with an adverse event that resulted in an unscheduled return to theatre; and surgical site infection rate: 29 (5.3%) operations were associated with an infection.

**Conclusion:**

Complications and adverse events are common in paediatric neurosurgery. Prospective, continuous surveillance will promote both quality assurance and quality improvement in the neurosurgical care delivered to patients.

## Introduction

The French vascular surgeon Rene Leriche [7] famously wrote:

“Every surgeon carries about him a little cemetery, in which from time to time he goes to pray, a cemetery of bitterness and regret, of which they seek the reason for certain of their failures”*.*

In recent years, there has been much greater scrutiny of adverse events in healthcare systems and a surgeon’s personal cemetery is no longer private but to be opened for public scrutiny.

Individual consultant surgeon- and institution-specific mortality rates have been published in the UK since 2014. When first published, the NHS Medical Director, Sir Bruce Keogh, said that surgeons had a moral responsibility to make public their death rates, in arguing that this was a means to defend how well they delivered a service; he directly linked surgical outcome data to quality and safety.

Morbidity and mortality should be recorded in a systematic way; in addition to recording any adverse event, the system must also define the denominator—how often could such an event have occurred, to determine the true rate. When comparisons can be made between units, the standard of care can be defined.

We sought to devise a system for the prospective surveillance of all morbidity and mortality in paediatric patients (<16 years) admitted to our hospital. Our system was based on the model devised by Drake and colleagues [5] but was adapted and extended to include a qualitative assessment of impact on the patient’s experience. We present our results following 2 years of data collection, propose three quality indicators that would permit ongoing monitoring of quality and comparison between units and review the current literature on morbidity in paediatric neurosurgical patients.

## Methods

The paediatric neurosurgical unit at the Royal Hospital for Children, Glasgow, is the largest unit in Scotland, and in addition to providing neurosurgical care to the population of the west of Scotland (population 2.7 million), it is one of the designated supra-regional national UK centres for craniofacial surgery. The unit has four consultant paediatric neurosurgeons, a specialist trainee, a fellow, an advanced nurse practitioner and a clinical nurse specialist.

The model devised is based on that proposed by Drake and colleagues [5], each adverse event categorised in the scheme outlined in Table 1.

**Table 1:** Classification of complications

Surgical	Neurological deficit	New deficit
	Meningitis	Excluding indwelling drain
	Seizures	
	Wound infection	
	CSF leak	
	EVD/lumbar drain infection	Suspected or confirmed
	Shunt blockage	
	Shunt infection	Suspected or confirmed
	Post-operative haemorrhage	Managed medically
	Post-operative haemorrhage	Returned to theatre
	Post-operative infarction	
	Other	
Medical	Cardiac	
	Respiratory	Inc pneumonia
	GI/hepatic	
	Renal/GU/UTI	
	Haematological/thromboembolic	
	Metabolic	
	Cognitive	
	Other	

To be included, an adverse event had to occur either during their inpatient stay or if discharged within 30 days of surgery. Wherever possible, the adverse event is linked to the appropriate neurosurgical operation.

Each potential adverse event is prospectively recorded and then discussed at a monthly review meeting (chaired by the unit clinical governance lead, EC). The adverse event is assigned a category and linked to the appropriate operation. An additional patient impact score was assigned using our organisation’s matrix for severity categorisation of complications [11] (Table 2).

**Table 2:** NHS Greater Glasgow and Clyde impact/severity descriptors

1	Negligible	Reduced quality of patient experience/clinical outcome not directly related to delivery of clinical care
2	Minor	Unsatisfactory patient experience/clinical outcome related to care provision—readily resolvable
3	Moderate	Unsatisfactory patient experience/clinical outcome; short term effects—expect recovery <1 week
4	Major	Unsatisfactory patient experience/clinical outcome; long-term effects—expect recovery >1 week
5	Extreme	Unsatisfactory patient experience/clinical outcome; continued ongoing long-term effects

If the adverse event resulted in the patient returning to theatre for a further procedure, the impact score was a minimum of 3. An adverse event with an impact score 3, 4 or 5 was termed significant.

A governance meeting, attended by the entire paediatric neurosurgical unit, with representation from allied medical and surgical specialties is held every quarter. At this meeting, each event is reviewed and the final decision whether it is to be included in the morbidity database, its category and impact score is agreed. All inpatient deaths are also reviewed. Multiple complications in the same patient were counted separately, and each was linked to the appropriate procedure, if the patient had undergone more than one procedure.

Three key indicators were identified to permit monitoring of quality of care delivered:

1. Significant adverse event rate—the proportion of operations to which at least one significant adverse event was linked to.
2. Unscheduled return to theatre rate—the proportion of operations which subsequently required an unplanned return to theatre due to a recorded significant adverse event.
3. Surgical site infection rate—the proportion of operations that were linked to any adverse event affecting the surgical site (wound, shunt or drain infections).

The database was recorded and analysed on Microsoft Excel, and chi-squared test was used to analyse categorical variables and a two-sample *t* test to analyse continuous variables. The project was approved by the local clinical governance support unit.

## Results

Data was prospectively collected from 1 January 2014 until the 31 December 2015 for all patient aged <16 years, admitted under the care of the paediatric neurosurgical team. Five hundred forty-nine procedures were performed in 287 patients (163 males, 124 females). The mean age at the time of the operation was 5.8 ± 5.0 years (mean ± standard deviation). One hundred thirty-five (24.6%) operations were performed in patients less than 1 year of age at the time of the operation.

One hundred thirty-eight (25.1%) operations were classified as routine; the remainder was non-elective (urgent and emergency procedures). The principal surgeon was the consultant paediatric neurosurgeon in 307 procedures (55.8%); the majority of the remainder of cases were performed by a trainee as the principal surgeon, supervised by the consultant paediatric neurosurgeon (228 procedures, 41.5%). A small minority of cases were performed by an adult consultant neurosurgeon (14 procedures).

Eight patients died during the study period, six of whom had undergone a neurosurgical procedure in our department during their last hospital stay. All deaths were formally reviewed at the quarterly governance meeting. In the review of two of the deaths, it was concluded that given the underlying diagnosis, the death was unexpected and the adverse events that had occurred during the final inpatient stay were likely to have contributed to the patient’s death. One patient (31 days old) suffered an intraventricular haemorrhage following embolisation of a vein of Galen malformation, an external ventricular drain was inserted, but the baby never recovered from the haemorrhage, and treatment was withdrawn. The second death occurred in a 2-year 3-month-old child who presented in coma with a posterior fossa tumour. Despite emergency surgery and excision of the tumour, she suffered a devastating hypoxic ischaemic brain injury and treatment was ultimately withdrawn; histopathology showed the tumour to be a pilocytic astrocytoma.

For the remaining six deaths, it was concluded that the death was expected and the result of the underlying disease process rather than any adverse event that had occurred.

The calculated crude mortality rate for patients undergoing neurosurgery in our department was 6/287 = 2.1%.

One hundred ninety adverse events were recorded; 130 had an impact score of 3 or greater and were termed significant. Table 3 outlines the category and impact score of the recorded adverse events. Thirty-one adverse events were medical, and 158 adverse events were surgical. The commonest adverse event was a CSF leak; the commonest significant adverse event was a shunt blockage.

**Table 3:** Frequency of adverse event by category and impact score

		Impact score, no. of events					Total
		1	2	3	4	5	
Surgical	Neurological deficit		3	3	12	1	19
	Seizures		5	1	2		8
	Wound infection		2	6	1		9
	Wound breakdown		1	1	2		4
	CSF leak		20	21			41
	EVD/lumbar drain infection		2	6	1		9
	Shunt blockage			27	3		30
	Shunt infection			7	4	1	12
	Post-operative haemorrhage (medical)		1	1			2
	Post-operative haemorrhage (surgical)				1	1	2
	Other^a^	4	7	8	3	1	23
Medical	Cardiac						0
	Respiratory			3	3		6
	GI/Hepatic		1				1
	Renal/GU/UTI			1			1
	Haematological/thromboembolic				1		1
	Metabolic	1	11	2	3		17
	Other		2	2		1	5
Total		5	55	89	36	5	190

^a^The 12 surgical, significant adverse events (impact score 3, 4 or 5) recorded under ‘other’ included a delay in treatment of raised ICP (1), injury to the small bowel during insertion of a ventriculoperitoneal shunt (1), pressure sore (2), EVD blocked (3), dislocated right elbow (1), ICP monitor or vascular long line dislodged (2) and failure to drain all ventricles following revision of VP shunt (2)

### Significant adverse event rate

One hundred eleven operations were linked to at least one significant adverse event, giving a significant adverse event rate of 20.2% (6/190 adverse events were not be linked to a neurosurgical procedure).

### Unscheduled return to theatre rate

Of the 549 operations performed, 81 were associated with an adverse event that resulted in an unscheduled return to theatre, 14.8%.

### Surgical site infection rate

Thirty operations were associated with a surgical site infection or a suspected infection for which treatment was instigated; the overall surgical site infection rate was 5.5% (30/549). The causative organism was identified in 23 of the 29 infections; 26 of the infections were classified as a significant adverse event.

CSF diversion operations had a higher significant adverse event rate (26.5%) compared to spinal operations and all other cranial procedures (21.9 and 12.2%, respectively) (*p* < 0.001). Within the subcategories of cranial surgery, tumour surgery had the highest significant adverse event rate, 32.1% (Table 4).

**Table 4:** Category of operation

Category of operation	Number of operations	Number of operations linked to significant adverse event	Significant adverse event rate (%)
Brain tumour	53	17	32.1
Epilepsy	8	0	0
Trauma	16	0	0
Craniofacial	48	3	6.3
Vascular	22	3	13.6
Other	83	5	5.6
All cranial	230	28	12.2
CSF diversion	287	76	26.5
Spine	32	7	21.9

Routine operations had a lower significant adverse event rate than non-routine operations (11.6 vs 23.1%, *p* = 0.004).

There was no real difference in the significant adverse event rate in operations where the paediatric consultant was the principal surgeon and where they were not (20.5 vs 19.8%, *p* = 0.84). There was no statistical difference in the proportion of male and female patients who experienced a significant adverse event following surgery (male 21.4 vs 18.6%, *p* = 0.42); nor was there any difference in the mean age of patients who experienced a significant adverse event (6.23 vs 5.71 years, *p* = 0.33).

## Discussion

In his recent book, ‘Black Box Thinking’ [17], Syed explores how success happens and analyses the approach that different industries and professions take when failure occurs. He draws many parallels between the aviation industry and healthcare; both are safety-critical, high-performance professions, which utilise increasingly complex systems, but he argues that there is a profound difference in how they approach adverse or unexpected events. Pilots and aviation system experts perceive such events as the consequence of the conflict between the complexity of the system and their capacity to understand it. In healthcare, Syed contends that too often, such events are seen not as the inevitable consequence of complexity but as an indictment of those who make them. Our development of a surgical morbidity surveillance system is the first step in this process of changing the culture to adverse events in our department.

### What is a surgical complication?

Dindo and Clavien [3] emphasised the distinction between the failure to achieve the surgical goal and a complication in their definition; “any deviation from the ideal post-operative course that is not inherent in the procedure and does not comprise a failure to cure.”

Sokol and Wilson [16] differed and argued that an undesired outcome, which would include a failure to cure, is perceived as a surgical complication; “any undesirable, unintended and direct result of an operation affecting the patient, which would not have occurred had the operation gone as well as could have been reasonably be hoped”.

If the goal of a prospective morbidity surveillance programme is to identify areas where surgical care could be improved, we argue that this should include when the primary goal of the operation has not been achieved. We sought to identify any adverse event that had occurred in patients admitted under our care and thus adopted the Sokol and Wilson definition that this would mean any clinical event that was undesired and unintended.

### Classification of surgical complications

The Clavien and Dindo classification [4] is the most widely cited classification system; designed for general surgery, its application has been extended to related surgical specialties, for example to urology, and Ibanez et al. [6] proposed a modified form of the system for neurosurgical patients. The Clavien-Dindo system classifies a complication according to the therapy used to treat it and thus is an assessment of impact severity; for example, wound infection requiring surgical washout and replacement of an external ventricular drain would both be grade IIb: complication requiring intervention with general anaesthesia. As it does not make a distinction between different categories of complication, its practical use as a means for improving clinical practice is more limited.

It is also important to remember that these systems were designed for adult patients; taking action after an adverse event in a child may require a greater level of intervention than in an adult; for example, resuturing a wound or performing a lumbar puncture may require a general anaesthetic in a child, but this is less likely in an adult; thus, the grade of the complication will have increased.

We adopted the system proposed by Drake and colleagues [5] for several reasons. Firstly, it was specifically designed for neurosurgical patients and the categorisation of the complication by surgical and medical subgroups would be easily applied in a busy paediatric neurosurgical unit. The same complication occurring with different procedures could more easily be identified. Secondly, the system has been adopted by other paediatric neurosurgical units [19], permitting a comparison between units. Lastly by linking each complication to an operation, the Drake system creates a simple and easily counted denominator. This gives a more accurate assessment of the significant adverse event rate than linking it to individual patients; for example, estimating the 30-day shunt blockage rate is more meaningful if we compare the number of shunts that blocked in 30 days to the total number of shunts inserted, rather than the total number of patients who underwent the procedure.

We modified the model to include an assessment of patient impact severity, using our organisation’s clinical risk matrix. Drake et al. [5] classified morbidity “as any significant adverse outcome or death”; we felt that the term significantly needed to be more precisely defined. Whilst our system is simpler than the Clavien-Dindo system, we believe that the dual categorisation of an event adds strength.

### Comparison of complication rates

Our significant adverse event rate was 20.2%; in comparison to other units using the same system, Drake et al. [5] reported an overall complication rate of 16.4% over 2 years and van Lindert et al. [19] reported an overall complication rate of 20.2%. Using a different system, Patel et al. [13] also reviewed all cranial and spinal surgery and published a 30-day morbidity rate of 15.0%. Taking into account differences in interpretation and data collection, we believe that our result that approximately one in five neurosurgical procedures resulted in an adverse event is in keeping with current published rates for paediatric neurosurgical units.

Moiyadi et al. [9] and Neervoort et al. [10] reviewed the morbidity associated with cranial operations for CNS tumours and identified morbidity rates of 44.4 and 68.6%, respectively. We found that our cranial operations for tumour have a significant adverse event rate of 32.1%; this trend of higher morbidity for tumour operations in comparison to the overall morbidity rate within a unit was also recorded by van Lindert [19], 32.7%, and Drake [5], 27.9%. This study supports the view that morbidity following tumour surgery is greater than other types of neurosurgery in paediatric patients.

In reviewing our significant adverse event rates, we identified that our significant adverse event rate (SAER) was greater for CSF diversion procedures, 26.5% (shunt insertion and revision, external ventricular drain insertion, endoscopic procedures and open craniotomy procedures for arachnoid cysts), than for other cranial procedures or spinal procedures. This appeared to be higher than that published by other units (van Lindert et al. [19] 21.8% and Drake et al. [5] 15.4%). In light of this finding, a detailed analysis of the CSF diversion procedures was completed. It was found that the SAER was highest for shunt procedures (71 new shunt insertions and 75 shunt revisions) and the commonest surgical complication was shunt blockage. Whilst it appeared that during the 2-year period of the study, we had at least three complex patients in whom it proved extremely difficult to maintain a working CSF drainage system, our department identified several areas where intraoperative technique could be modified with the aim of reducing the frequency of early shunt failure.

We reported an unscheduled (30 days) return to theatre rate of 14.8%; this excluded those patients who had a planned return to theatre for a planned second operation, for example tumour resection after a first operation to treat hydrocephalus.

A rate of 14.8% is in keeping with the rates published by other paediatric neurosurgical units; Van Lindert and colleagues [19] reported their unscheduled return to theatre rate of 10.5%; Mukerji and colleagues [9] reported a 30-day unplanned reoperation rate of 17%.

Mukerji et al. advocated the adoption of an unplanned reoperation rate as a quality indicator; we agree with their view that it is an easily counted, common event that is non-discretionary. They reviewed all reoperations in a 2-year period and found that the majority of the reoperations (61%) occurred within 30 days of the original operation (median time between first and unplanned reoperations was 9 days).

The necessity in identifying the causative organism in a surgical site infection has been extensively debated. We argued that if there was a clinical suspicion of an infection, for example, a child with symptoms of meningitis, a fever and a raised white cell count in their CSF following a shunt revision, the failure to identify the causative organism did not delay or defer treatment; therefore, any child who underwent treatment of an infection should be defined as having an infection.

We linked 29 operations to a surgical site infection: 5.3% of all procedures. Interestingly, the causative organism was identified in 22 of these infections, which if we had used the criteria of identification of the causative organism, employed by other units, would have given a surgical site infection rate of 4.0%.

These rates of surgical site infection are in keeping with that published by other units for both all types of neurosurgery and specific subtypes (Table 5).

**Table 5:** Literature review of published morbidity and mortality studies in paediatric neurosurgical patients

Study	Age of patients (years)	Duration of observation (days)	Type of surgery	Number of patients	Number of procedures	Morbidity (%)	Mortality (%)	30-day reoperation rate (%)	Surgical site infection rate (%)
Albright [1]	NS	Varies	Shunt insertion/shunt revision/craniotomy for brain tumour/craniotomy for sagittal synostosis/tethered cord	200/72/100/91/100	200/72/100 /91/100	NS	1% for brain tumours	NS	7/11/4/0/9. Composite infection rate, 6.6
Smith [14]	0–18	Inpatient stay	Cranial—tumours only	4712	NS	NS	1.60	NS	NS
Drake [5]	NS	30 days	All cranial and spinal surgery	NS	1082	16.4	11 deaths	10.4 (estimated)	3.1 (estimated)
Neervoort [10]	0–18	NS	Cranial—tumours only	121	137	68.6	0.8	NS	NS
Moiyadi [8]	0–18	NS	Cranial—tumours only	117	117	44.4	7.7	Reexploration, 8.5; additional procedure, 14.5.	15.4
Murkerji [9]	NS	300 days	All cranial and spinal surgery	NS	NS	NS	2.7	17.3	NS
Solheim [15]	0–16	30 days	Cranial—tumours only	1242	1242	NS	3.1	NS	NS
Von Lehe [18]	0–16	30 days	Cranial	641	769	2.0	NS	NS	3.9
O’Kane [12]	0–14	30 days	Cranial—tumours only	1280	NS	NS	2.7	NS	NS
Patel [13]	NS	30 days	All cranial and spinal surgery	1990	3195	15.0	1.6	NS	NS
Van Lindert [19]	0–17	30 days	All cranial and spinal surgery	581	1000	20.2	2.2	10.5	3.4
Desai [2]	NS	30 days	Emergency and urgent surgery	580	710	10.3	5.2	4.7	3.3
Van Lindert [20]	0–17	Intraoperative only	All cranial and spinal surgery	1148	1807	3.5	0.2	NS	NS
Current study	0–16	30 days	All cranial and spinal surgery	287	549	20.2	2.1	14.8	5.5 (4.2% organism identified)

*NS* not specified

## Conclusion

Whilst most paediatric neurosurgery centres review their morbidity and mortality, we believe that our unit’s surveillance model has formalised this process and permits an ongoing method of both quality assurance and quality improvement in the care that we provide for our patients.

The model that we have adopted was specifically designed for paediatric neurosurgery, and our experience has been that it can be implemented into a busy unit with minimal disruption of day-to-day clinical activity.

The proposed three key indicators will provide a means for comparison between units for both overall and subcategories of surgery, thus further promoting quality assurance within our speciality.
